# Restricted Speech Recognition in Noise and Quality of Life of Hearing-Impaired Children and Adolescents With Cochlear Implants – Need for Studies Addressing This Topic With Valid Pediatric Quality of Life Instruments

**DOI:** 10.3389/fpsyg.2019.02085

**Published:** 2019-09-12

**Authors:** Maria Huber, Clara Havas

**Affiliations:** ^1^Department of Otorhinolaryngology, Head and Neck Surgery, University Hospital Salzburg, Paracelsus Medical University, Salzburg, Austria; ^2^Department of Pediatrics, University Hospital Salzburg, Paracelsus Medical University, Salzburg, Austria

**Keywords:** QoL, hearing loss, pediatric cochlear implantation, speech recognition in noise, valid instruments

## Abstract

Cochlear implants (CI) support the development of oral language in hearing-impaired children. However, even with CI, speech recognition in noise (SRiN) is limited. This raised the question, whether these restrictions are related to the quality of life (QoL) of children and adolescents with CI and how SRiN and QoL are related to each other. As a result of a systematic literature research only three studies were found, indicating positive moderating effects between SRiN and QoL of young CI users. Thirty studies addressed the quality of life of children and adolescents with CI. Following the criteria of the World Health Organization (WHO) for pediatric health related quality of life HRQoL (1994) only a minority used validated child centered and age appropriate QoL instruments. Moreover, despite the consensus that usually children and adolescents are the most prominent informants of their own QoL (parent-reports complement the information of the children) only a minority of investigators used self-reports. Restricted SRiN may be a burden for the QoL of children and adolescents with CI. Up to now the CI community does not seem to have focused on a possible impairment of QoL in young CI users. Further studies addressing this topic are urgently needed, which is also relevant for parents, clinicians, therapists, teachers, and policy makers. Additionally investigators should use valid pediatric QoL instruments. Most of the young CI users are able to inform about their quality of life themselves.

## Introduction

A cochlear implant (CI) is a prosthesis for the hair cells in the inner ear for individuals with severe to profound hearing loss. CIs support the development of oral language in hearing-impaired children (e.g., Geers et al., 2016; Cupples et al., 2018; Ruben, 2018), so that children with bilateral CIs perform better than children with unilateral CI (Lovett et al., 2010; Geers et al., 2016; Moberly et al., 2016; Health Quality Ontario, 2019). However, there are limits to CI. Not all children with CI develop language at an average level (e.g., Sarant, 2012; Geers et al., 2016; Lund, 2016; Cupples et al., 2018). Geers et al. (2016) found a persistent language delay in 32% of 10.5 year old children with CI. Furthermore, children with CI are more restricted in speech recognition in noise (SRiN) compared to their normal hearing peers (Caldwell and Nittrouer, 2013; Chen et al., 2014; Taitelbaum-Swead and Fostick, 2017). SRiN depends on the language abilities of young CI users (Ching et al., 2017), duration of CI use, education of the mother, use of hearing aids before CI, pre-implant auditory threshold (Chen et al., 2014) and bilateral CI (vs. unilateral CI, Lovett et al., 2010; Sparreboom et al., 2012; Jacobs et al., 2016). Additionally, in the case of normal hearing children SRiN depends on their cognitive abilities (Roman et al., 2017).

In our noisy world, the ability to recognize and to understand speech in noise is of tremendous importance. Overall noise pollution may have more profound effects on children than on adults, because their cognitive functions are “less automatized and thus more prone to disruption” (Klatte et al., 2013). Furthermore, children have fewer options to influence their environment. Nevertheless, it seems that children are often exposed to substantial noise. Indoor noise levels in playschools and schools are often higher than the recommended maximum noise levels (Sarantopoulos et al., 2014; Chan et al., 2015). For example, the indoor noise level in occupied classrooms was on average 69 LAeq dB^1^ and in unoccupied classrooms 47 LAeq dB (Sarantopoulos et al., 2014). The level of speech was estimated to be only 12 dB higher than the level of background noise (speech-to-noise ratio) during teaching and even less discernible during break time and outdoor activities (Sarantopoulos et al., 2014). Noise has a negative impact on school performance of normal hearing children. The performance on working memory tasks and comprehension tasks is impaired during lessons with indoor noise (Klatte et al., 2013; Sullivan et al., 2015).

Studies about hearing-impaired children indicate that restricted SRiN compromises not only the hearing health and functioning (listening and understanding), but also other areas of physical, mental and social health [see the health concept of the World Health Organization [WHO], 1948]. The effort in SRiN for hearing-impaired students is shown in longer reaction times in verbal tasks (*impeded physical health*) compared to normal hearing peers (McGarrigle et al., 2019). FM systems (Frequency Modulation radio waves send speech and other auditory signals to hearing aids or CI) support hearing-impaired students during lessons. However, not all students are using them continuously during lessons (Keilmann and Reutter, 2014). Restricted SRiN is also associated with physical stress (*physical health*), as indicated by elevated cortisol levels (Bess et al., 2016) and by fatigue (Hornsby et al., 2017). Parents seem to underestimate the fatigue of their children with CI, which may be disappointing and frustrating for the children and might lead to feelings of isolation (*impeded social health*, Werfel and Hendricks, 2016; Hornsby et al., 2017). Furthermore, restricted SRiN correlated positively with internalizing and externalizing problems of adolescent CI users (*impeded mental health*, Huber et al., 2015) and may be one of the reasons, why young CI users have more peer problems (*impeded social health*, Huber et al., 2015; Warner-Czyz et al., 2018). Accordingly, the question arises, if restricted SRiN impedes the subjective wellbeing of hearing-impaired children and adolescents, growing up with cochlear implants.

Subjective wellbeing (SWB) can be understood as a “summary measure of quality of life” (Wilson and Cleary, 1998) and is usually characterized by three domains: (i) positive affect, (ii) life satisfaction and (iii) meaning and purpose of life (Ravens-Sieberer et al., 2014a; Wallander and Koot, 2016). Quality of life (QoL) concerns different life areas like the individual‘s economic status, rights, culture and health (Fayed et al., 2012). Health related quality of life or HRQoL is commonly “considered to be a subdomain of the more global construct of QoL” (Davis et al., 2006). Based on the health concept of the World Health Organization [WHO] (1948) HRQoL spans the domains *physical health*, *mental health*, and *social health*. However, there is a lack of a common definition of pediatric QoL (HRQoL and SWB), see e.g., Drotar (2004), Davis et al. (2006), Fayed et al. (2012), Ravens-Sieberer et al. (2014a, b), and Wallander and Koot (2016).

For the assessment of pediatric QoL, child specific instruments are needed. According to the World Health Organization (WHO) valid pediatric QoL measures should be (i) *child-centered*, i.e., specifically developed for children, (ii) *age-appropriate*, taking into account the developmental status of different age groups, (iii) *validated* cross-culturally, and (iv) include *self-reports* (World Health Organization [WHO], 1994). Regarding (i) and (ii) recent studies demonstrate a downturn of SWB (Wallander and Koot, 2016) and HRQoL in adolescence (Warner-Czyz et al., 2011; Rajmil et al., 2013; Barkmann et al., 2016; Raj et al., 2017). Therefore, specific self- and parent reports for different age groups should be available. Regarding (iii) a consensus exists that children and adolescents are the most prominent informants of their own QoL (Riley, 2004; Davis et al., 2006; Upton et al., 2008; Ellert et al., 2011; Ravens-Sieberer et al., 2014a, b).

Children at the age of 5 years are able to inform about their health states, health functioning (Riley, 2004) and SWB (Ravens-Sieberer et al., 2014a). From the age of eight on, children are able to report reliably “on all aspects of their health experiences and can use a five-point response format” (Rebok et al., 2001). However, reports are only possible with “child friendly questionnaires” (Coghill et al., 2009). This implicates, that the questions of the self-report correspond to the language level, speech style, reading skills and cognitive status of the respective age-group (Rebok et al., 2001; Riley, 2004; Davis et al., 2006; Coghill et al., 2009; Ravens-Sieberer et al., 2014b). Parent reports should complement the reports of the children, completing “the picture of a child’s QoL” (Coghill et al., 2009). Children can be too young or unable to understand the questions, for example because they have additional special needs. In this case parent reports are not only required, but essential. Most studies showed only a poor to moderate agreement between parent reports and children’s reports about the child’s QoL (Eiser and Varni, 2013; Rajmil et al., 2013; Silva et al., 2015; Lee et al., 2019. See however Quitmann et al., 2016). Accordingly, the question arises, whether QoL outcomes in CI users vary, depending on whether parent or self-ratings are considered.

Validated SWB and HRQoL instruments for children and adolescents correspoding the criteria of the WHO are listed in Davis et al. (2006), Fayed et al. (2012), Ravens-Sieberer et al. (2014a, b), and in Wallander and Koot (2016).

**FIGURE 1:**
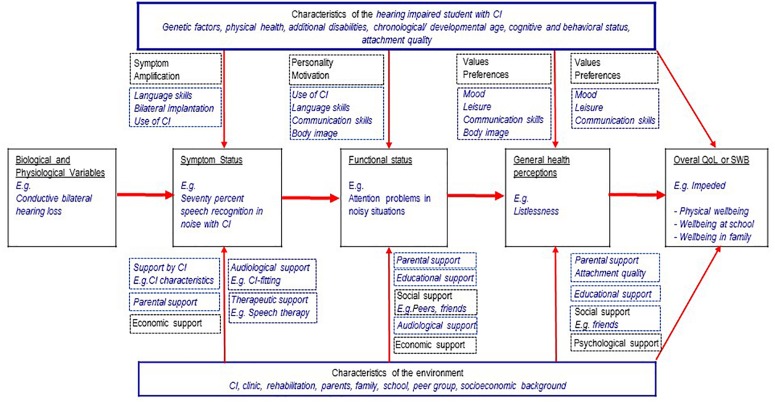
Wilson and Cleary theoretical framework (1998, 10.1371/journal.pone.0113166.g001) adapted to young CI users, with permission of the authors and the copyright holder (4/10/19, 4565201160405).

The model of Wilson and Cleary (1998) is one of the most prominent and best validated models of HRQoL in adults (Bakas et al., 2012; Ojelabi et al., 2017). According to this model, biological/physiological variables (“cells, organs, and organ systems”) influence the symptom status, e.g., fever. The symptoms in turn influence the functional status, e.g., some gross motor activities (“Measures of function assess the ability of the individual to perform particular defined tasks.”). This again has an impact on the (subjective) general health perceptions (a “subjective rating” of one’s own health), and finally the “overall quality of life” (QoL), e.g., worry because of a disease. Additionally, there are individual influences (personality, motivation, preferences, and values) and environmental influences (social, economic and psychological support of the environment). Figure 1 shows an adaptation of this model illustrating an example of the HRQoL of a young CIs user with a congenital hearing loss. In this example, restricted SRiN may cause attention problems (functioning) and listlessness (health perception). Possible consequences may be impeded physical wellbeing and aggrieved wellbeing at school, see Figure 1.

There may be other situations causing reduced QoL in young CI users with cascading effects: Communication problems with peers, caused by restricted SRiN may provide the perception of isolation and impede the social wellbeing. If parents do not notice the problems of their child, this may impede the wellbeing of the child in the family. If restricted SRiN is associated with more internalizing and externalizing problems, these problems may impede the mental wellbeing. Problems to follow instructions at school or at training, caused by restricted SRiN may worsen the appraisal of the teacher. This is possibly perceived as failing by the CI user and may impede the wellbeing at school or at the vocational place. In all these situations, there may be mediating effects between SRiN and QoL. There can also be direct effects: A young CI user perceives problems to follow the conversation because of the background noise at a party and is impeded in his social wellbeing. Studies in normal hearing children showed correlations between HRQoL and fatigue of children with cancer (Nunes et al., 2017), internalizing and externalizing problems (Dey et al., 2012; Ravens-Sieberer et al., 2012) and academic performance (Degoy and Berra, 2018).

To address the question, whether SRiN impedes HRQoL in children and adolescents a systematic review was intended strictly following the PRISMA criteria (Liberati et al., 2009). However, the systematic literature research resulted in only three papers. Therefore, we did not carry out a meta-analysis, and present the findings of our research in the format of a perspective article. In a first step, we identified all papers reporting about the HRQoL in young CI-users and summarize the outcomes, to address whether QoL was impaired in young CI-users. In a second step, we summarize the three papers reporting a relationship between SRiN and HRQoL in young CI-users, which are the main focus of this article.

## Materials and Methods

The procedure strictly followed the PRISMA statements. Included were papers addressing SRiN measured with speech recognition tests and QoL, HRQoL, or SWB of children and adolescents with CI, as primary or secondary outcome. HRQoL or SWB was measured with validated child-centered and age-appropriate QoL instruments (see Supplementary Material for inclusion criteria). Papers about CI users with single sided deafness were not included. We considered publications until January 2019 (see Supplementary Material for the search terms and review procedure). Primary outcomes were the correlation between SRiN performance and QoL of children and adolescents with CI and the improvement of QoL after an improvement of SRiN, respectively. The risk for biases was estimated with a short checklist (orientation to the Cochrane risk of bias tool, Cochrane Deutschland, 2016, see also Supplementary Material).

## Results

In step 1 of our review, we removed 1851 citations, all papers that were not in English or not original research articles. In step 2 191 citations were removed, which reported studies in adults, very young children, cost utility analyses or represented a double citation. The remaining 32 papers were reviewed. Two papers were removed, because no original data about QoL, HRQoL, or SWB of young CI users were reported, or the data were already reported elsewhere (see the flow diagram in the Supplementary Material). Only eleven out of the remaining 30 studies, i.e., one third, used validated child-centered and age appropriate QoL instruments, see Table 1 and Supplementary Material. 19 studies did not use valid pediatric QoL instruments^2^. Additionally, as summarized in Table 1, 20 out of 30 studies relied exclusively on parent or teacher reports with a trend toward more positive QoL results, compared to the 10 studies relying additionally on self-reports, see Table 1.

**TABLE 1:** Studies addressing quality of life of children and adolescents with CI.

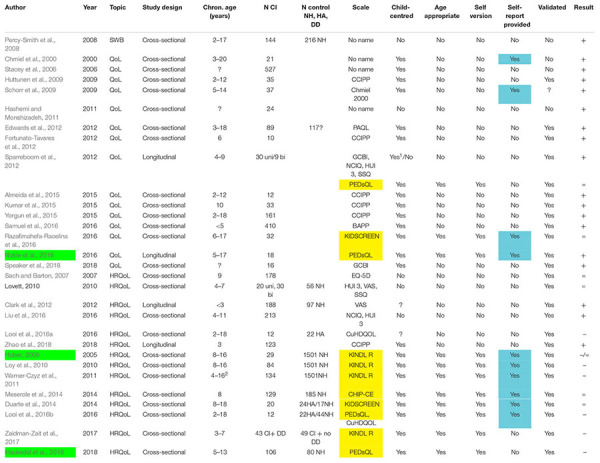													

*QoL, Quality of life; HRQoL, Health Related Quality of Life; SWB, Subjective Well Being; mo, months; c, children; p, parents; t, teachers; CI, CI users; NH, normal hearing; uni, unilateral; bilat, bilateral; HA, hearing aid; DD, developmental disability; Self version, Instrument with specific self versions; Self-report provided, young CI users were asked; EQ-5D, EuroQoL; CCIP, Children with cochlear implants parental perspectives; HUI, Health Utility Index; VAS, Visual Analog Scale; PAQL, Paediatric Audiology Quality of Life; NCIQ, Nijmegen Cochlear Implantation Questionnaire; GCBI, Glasgow Children Benefit Inventory; SSQ, Speech, Spatial, and Qualities of Hearing Scale; BAPP, Brief Assessment of Parental Perception; CuHDQOL, Children Using Hearing Devices Quality of Life Questionnaire (?); KINDL R, KINDL revised https://www.kindl.org/; CHIP-CE, Child Health and Illness Profile-Child Edition, https://www.ideaconnection.com/patents/5679-Child-Health-Illness-Profile-Child-Edition-CHIP-CE-Ch.html; KIDSCREEN, Health Related Quality of Life Questionnaire for Children and Young People and their Parents; https://www.kidscreen.org/; PEDsQl, Pediatric Quality of Life Inventory, http://www.pedsql.org/.(?) not clear; (+) Improved QoL in CI-group after CI, positive Qol after CI, improved QoL after remapping, satisfaction of parents with QoL of children with CI, Children with Equal or better scores of children with CI compared to normal-hearing peers (=) No Difference between pre and post CI (evaluation of status pre CI retrospectively post CI), No difference between unilateral and bilateral CI, No change in QoL after use of CI, No difference between unilateral and bilateral CI, No difference between adolescents with CI and normal hearing peers, No difference between CI group and Normal Hearing group, No difference between CI group and norm group of normal hearing peers. (–) Lower Qol of CI users, compared to HA users, Lower QoL of CI users (at least in some domais), compared to normal hearing peers, Lower Qol with growing age, Lower QoL of CI users with developmental delay compared to CI users without delay.*

*Yellow background: Paper with validated child centered, and age appropriate QoL, instrument (SWB, HRQoL).*

*Green background: Papers addressing the association between QoL measured with a child-specific self-rating scale and SRiN as examined with audiological speech recognition tests.*

*Blue background: Papers using self-reports. ^1^GCBI. ^2^Samples of Loy et al. (2010) and Warner-Czyz et al. (2011) may be partially identical.*

In a small retrospective study, Huber (2005) addressed the HRQoL of 18 children with CI (at average 10.7 years old) and 12 adolescents with CI (at average 14.4 years old). There was a moderate correlation between the SRiN performance and the HRQoL total score, but only in the self-rating of the children (Spearman’s *r* = 0.45, *p* = 0.03). Noble et al. (2016) performed a cohort study with 18 young CI users at average 10.7 years old. The authors investigated, if significant improvements in speech recognition in quiet and in noise (result of a remapping^3^) after 4 weeks were accompanied by an improvement in HRQoL. As the hearing performance in quiet and in noise of the CI users improved (*p* < 0.05), the HRQoL total score improved also (*p* < 0.05), but only in the self-rating. Haukedal et al. (2018) retrospectively compared the HRQoL parent rating of 106 CI users (mean age 9.2 years) with 80 normal hearing children and adolescents (mean age 9.3 years). CI group and NH group did not differ significantly in age; however, the IQ was significantly higher in the NH group. The authors found small correlations between scores on SRiN and the HRQoL total score (*r* = −0.28, *p* = 0.024), and the school functioning (*r* = −0.244, *p* = 0.048). However, the correlations did not survive controlling for age.

In summary, all three studies point to SRiN as a possible moderator of HRQoL in children and adolescents with CI. However, the correlations were only low, and none of these studies has examined the possible moderating relationship directly in a longitudinal design. Furthermore, two of the studies were underpowered and the third one had problems with the matching criteria between study and control group (higher IQ). One study was performed without self-reports. In all three studies the risk for biases (orientation to the Cochrane risk of bias tool, Cochrane Deutschland, 2016) was low to medium (compare Supplementary Material).

## Conclusion and Viewpoints

In a systematic literature research, we identified only three papers indicating positive moderating effects between SRiN and QoL of young CI users. This is astonishing, because numerous studies on adults with CI are dealing with this topic. In a systematic review and meta-analysis McRackan et al. (2018) listed 27 papers informing about 1394 adult CI users and found small, but significant associations (*r* = 0.24–0.26) between SRiN and HRQoL in adults. However, the results about hearing-impaired adults are not valid for hearing-impaired children and adolescents. First, because there is a difference in the onset of hearing loss: Most adults become hearing-impaired in adulthood, whereas most children and adolescents are hearing-impaired since birth. Second, because there is a difference in the living conditions of children and adults. For example, children must attend schools (in noisy schools, see above) whereas adults have more options to influence their environment and work place. Accordingly, there is an urgent need for further studies addressing possible associations between SRiN and QoL of young CI users.

Restricted SRiN may be a burden for the QoL of children and adolescents with CI. Information about this topic may also be relevant for parents, clinicians, and therapists who are usually not aware of a possible load of young CI users. Additionally, these studies are relevant for teachers and for policy makers, who are handling possible additional special needs, for example classes with fewer students or an additional support for trainees with CI in a noisy work place.

One of the reasons for this lack of studies may be a limited awareness of parents and clinicians for possible quality of life problems of hearing impaired children and adolescents with CI. Papers relying exclusively on parent/teacher ratings indicate a tendency toward more positive conclusions than the papers relying additionally on self-reports and may overestimate the QoL in young CI users. However, it remains to be clarified whether parents actually overestimate the QoL of their children with CI [see Huber (2005, 2007) and Loy et al. (2010) on one side and the Haukedal et al. (2018) on the other side]. Parents of non-clinical samples report higher child QoL than the children themselves (Upton et al., 2008).

Given these results, it was striking, that only a minority of studies on QoL in young CI users provided children’s self-reports. One problem that may arise with children’s self-reports are the language comprehension skills of children with CI. One should concede, that until school age many children with CI have a language delay (Sarant, 2012; Geers et al., 2016; Lund, 2016), but that the majority of older children and adolescents with CI have normal (age appropriate) language skills (Geers et al., 2016). Therefore, following the concepts of QoL (see section “Introduction”), we strongly recommend the inclusion of self-reports for school-aged children with CI. Additionally, speech tests examining lexicon and syntax can help to identify patients with insufficient language comprehension. If needed, written and oral support can be provided during the survey, which does not replace the questionnaire. However, these adaptions are only possible with the permission of the authors of the QoL instrument^4^.

Furthermore, it was striking, that the majority of investigators used non-valid instruments for the assessment of pediatric quality of life, which may bias the results. Some authors argued that the use of adult questionnaires or homemade questionnaires was justified for children and adolescents with cochlear implants, because there are no disease (problem) specific QoL instruments^5^ available. However, we think, that this no longer applies. The child HEAR-QL (Hearing Environments and Reflection on Quality of Life, 7–12 years, Umansky et al., 2011) and the adolescent HEAR-QL questionnaire, 12–18 years (Rachakonda et al., 2014) are validated problem specific HRQoL instruments. To our best knowledge these instruments are still waiting for a study addressing the HRQoL of young hearing impaired CI users. Using age-appropriate instruments is particularly important when obtaining self-reports.

### Final Conclusion

The question whether restricted SRiN impairs QoL of young CI users has been understudied, possibly due to an under-estimation of QoL problems in children and adolescents with CI. In order to adequately assess QoL in young CI-users, both parent- and self-reports need to be considered and valid pediatric QoL instruments should be used. Subjective well-being is an important component of QoL and the majority of young CI users are able to provide self-reports.

## Author Contributions

MH developed the proposal for the review, performed the review together with CH, wrote the draft version of the manuscript, and agreed to be primarily accountable for all aspects of the work. CH reviewed abstracts and articles for this review and adapted the draft version of this manuscript together with MH.

## Conflict of Interest Statement

The authors declare that the research was conducted in the absence of any commercial or financial relationships that could be construed as a potential conflict of interest.

## Footnotes

1 LAeq A – weighted equivalent continuous sound level.

2 Two thirds (19 papers) used adult QoL questionnaires, e.g., Nijmegen Cochlear Implantation Questionnaire, homemade questionnaires (e.g., Chmiel et al., 2000), and other PROMS for children (e.g., Children with cochlear implants parental perspectives), and adults (Speech, Spatial, and Qualities of Hearing Scale), see Table 1.

3 “Mapping (or MAPping) is the term for programming a cochlear implant to the specifications and needs of its use” (http://cochlearimplantonline.com/site/mapping-a-cochlear-implant/).

4 Support is provided by short standard sentences, which are presented both orally and written. Theses sentences are strictly following a written guideline. Paraphrase the questions of the survey, however, does not replace them. Please note, that permission of the authors of QoL instrument may be needed for this adapted format. In some cases the use of a sign language interpreter may be required.

5 In contrast to generic instruments.
