# Supplementary material for: Restricted Speech Recognition in Noise and Quality of Life of Hearing-Impaired Children and Adolescents With Cochlear Implants – Need for Studies Addressing This Topic With Valid Pediatric Quality of Life Instruments
Source: Front Psychol. 2019 Sep 12;10:2085. doi: 10.3389/fpsyg.2019.02085 (PMC6751251; doi:10.3389/fpsyg.2019.02085)
Supplement: Supplementary file 1 [file Data_Sheet_1.docx]

**Huber M, Havas C. Review, Information to the methods**

We performed this systematic review strongly following the criteria of the “PRISMA statement for reporting systematic reviews and meta-analyses of studies that evaluate healthcare interventions” (Liberati et al 2009). Eligibility criteria, information sources, search strategy, study selection and other methods of the analysis were defined in advance and described in an informal protocol.

The inclusion criteria are listed in **Table a.** We focused on children older than four years, because to our best knowledge there are no HRQoL (health related quality of life) or SWB (subjective well being) self-versions available for younger children (criterion (c)). Criteria (e) (f) (g) are based on the specific WHO standards for children (see manuscript). Criterion (h) is based on the general standards for HRQoL (see manuscript),

For example, the HRQoL instrument PEDsQL (Pediatric Quality of Life Inventory, **Varni et al. 2006**) is designed and validated for children, there are specific versions for younger and older children and for adolescents, and there are self-reports. Therefore the PEDsQoL corresponds to the criteria g-h. The PROM (Patient Reported Outcome Measure) GCBI (Glasgow Child Benefit Inventory, **Kubba et al. 2004**) is designed for children as well. However, there are no specific age versions and there are no self-reports. Therefore, the GCBI it does correspond to criterion (e), however, not to criteria (f) and (g).

We determined, that at least one HRQoL or SWB questionnaire used in the study had to fulfill criteria (e), (f), (g) and (h) for QoL instruments (**Table a**). Furthermore, studies remained included in the review, if it was only their secondary aim and not their primary aim to investigate the relation between SRiN and QoL (HRQoL or SWB) of children and adolescents with CI. Studies about children and adolescents with single sided deafness were excluded from this review. Furthermore, studies with samples of hearing impaired children and adolescents with and without CI were excluded, if no specific QoL results for children with CI were reported in the paper. And finally, cost utility analyses were excluded, if no QoL outcomes were reported, for example the HRQoL index score. Health utility measures are special HRQoL instruments mainly used for the economic evaluation of treatments.

The literature research was conducted in PubMed, Science Direct and Web of Knowledge (Thomas Reuters). We considered the publications until January 2019.

We used combinations of the following search terms: *“Speech perception in noise”/ Speech recognition in noise ” /”listening/hearing in noise”, “Quality of life”, “Health related quality of life”, “Subjective well-being” “QoL”, “HRQoL”, “SWB” “cochlear implant”, “pediatric cochlear implantation”, “children”, “adolescents”.*

Study selection: The review procedure consisted of the following three steps, which are illustrated in **Fig a**. After the keywords were entered in the search engines the aim of *Step 1* was to collect all abstracts, corresponding to criteria (a) and (b), see **Table a**. In the case of some doubt, the investigators skimmed through the abstracts. Aim of *Step 2* was to collect the papers, fulfilling at least criteria (a), (b), (c), (d) see **Table a**. In case of doubt, the investigators skimmed through the papers. Aim of *Step 3* was to find all papers fulfilling all criteria of the review, see **Table a**. This was done in two sub steps: Aim of *Step 3a* was to find all published papers reporting about QoL, HRQoL and SWB of young CI users, assessed with HRQoL instruments, validated for children and adolescents (fulfilling criteria a-i) see **Tables a** and **Fig a**). Aim of *Step 3b* was to find all published papers fulfilling all criteria, see **Tables a** and **Fig a**.

Candidate articles were independently reviewed by the authors familiar with the subject material and work in the field of CI. The reviews in Step 1 and Step 2 were performed by both reviewers together. The review in Step 3 was done independently by the reviewers. Hereby data extraction sheets and short checklists were used. In case of doubt additional literature research had to be performed.

The extraction of relevant data from each included study was done with self-created data extraction sheets. We extracted data informing about: a) number and characteristics of participants, including age, unilateral/bilateral CI, age at CI, years with CI, b) speech recognition in noise (SPiN) measure, including name, type of measure (pediatric or not), type of noise and results (e.g. speech in noise ratio in means, SD), c) QoL measure, including name, type of measure (generic, disease specific), type of rating (self-rating and or parent rating) and results (e.g. means, SD), and d) association between SRiN outcomes and QoL outcomes, including type of relationship (correlational relationship, causal relationship), statistical method (e.g. Pierson correlation) and effect size, e)study design.

Primary outcome was the correlation between SPiN performance and QoL of children and adolescents with CI, or the improvement of QoL after an improvement of SPiN.

To estimate the risk of bias of the included studies we constructed a short checklist (orientation to the **Cochrane risk of bias tool, Cochrane Deutschland 2016**) with the answer possibilities yes, no, and not clear, see more in the result section. This rating was performed independently by the two reviewers. In case of doubt, the viewers discussed the results until consensus.

We planned a meta- analysis, but did not carry out it due to small numbers of papers informing about this topic.

**Huber M, Havas C. Review, information to the results**

Study selection

Information about all exclusions of the review are listed in **Fig a**.

Four papers provided data about QoL and SRiN . The paper of **Sparreboom et al. (2012**) was removed, because the QoL measures used to evaluate the association between SRiN and QoL were no validated child-centered and age-appropriate instruments.

As a final result three papers of interest remained (**Huber 2005, Noble et al 2016, Haukedal 2018**).

Results of the included studies

In a small retrospective study, **Huber (2005)** addressed the HRQOL of 18 children with CI (at average 10.7y old, age at CI at average 4.3y) and 12 adolescents (14.4y old, age at CI at average 7.6y) see also **Table b**. For the examination of SRiN the HSM Sentence Test (**Hochmair, Schulz, Moser**) was used, an open set test (**Hochmair-Desoyer** et al. 1997). For the evaluation of the HRQoL the Kindl R was used, a generic (no disease specific) pediatric HRQoL (**Ravens-Sieberer et al. 2008**). There was as a significant and moderate correlation between the SRiN performance and the HRQoL total score, but only in the self-rating of the children (children self: Spearman’s r=0.45, p=0.03, children parents, adolescents self and parents: all rs<0.1, all p>0.05). There was no significant correlation between HRQoL total score and the variables chronological age, age at implantation, speech recognition in quiet (words) years of deafness, and years with CI (all p<0.05).

**Noble et al. (2016)** performed a cohort study with 18 young CI users at average 10.7 years old (no data about age at CI, see **Table b**). The authors investigated, if significant improvements in speech recognition in quiet and in noise (result of a remapping, see Footnote 2 in manuscript) after four weeks were accompanied by an improvement in HRQoL. For the examination of SRiN the BabyBio (**Spahr et al. 2014**), and the BKB-SIN (Bamford-Kowal-Bench**, Bench et al 1979**) were used, both pediatric open set sentence tests. For the evaluation of the HRQoL the PEDsQL (**Varni et al. 2006**) was used, a generic pediatric HRQoL instrument. As the hearing performance in quiet and in noise of the CI users significantly improved, the HRQoL total score (children) and “emotional functioning” (children, parents) improved also significantly (p<0.05). All other HRQoL differences were not significant. A statistical evaluation of the presumed association between improved SRiN outcomes and improved HRQoL was not performed.

**Haukedal et al. (2018)** compared retrospectively the HRQoL, parent- rating of 106 CI users (mean age 9.2 y, CI age of the “prelingually deaf” children on average 20 months, CI age of the other hearing impaired children on average 34 months) with 80 normal hearing children and adolescents (9.3y), see also **Table b** CI group and NH group did not differ significantly in age, however, the IQ was significantly higher in the NH group. Accordingly, matching was a problem in this study. SRiN was examined with the Norwegian adaptation of the Hearing in Noise Test (HINT) for children (**Myhrum et al. 2016**). For the assessment of HRQoL a Norwegian parent versions of the PEDs for younger children (5–7 years old) and children (8–12 years old) were used. The authors found small significant correlations between scores on the HINT and the HRQoL total score (r=−0.28, p=0.024), and the school functioning (r=−0.244, p= 0.048) „indicating that children who heard better in noise also had higher scores of HR-QOL in these domains“. However, the correlations did not survive controlling for age, suggesting again that the functional relationship between SRiN and HRQoL may differ between younger children and adolescents. There was no significant correlation between HRQoL scores and the variables, age at diagnosis of hearing loss, age at implantation, chronological age, nonverbal IQ, communication mode, speech recognition in quite (words), or social economic background (education of mother and father)(all p>0.05).

Risk of bias within studies

We rated the risk of biases for each paper, see also **Table c**

In **Huber (2005)** the risk for biases (orientation to the **Cochrane risk of bias tool, Cochrane Deutschland 2016**) was low in the selection of the participants, and in the evaluation of the endpoints (SRiN and HRQol). However it was high in the study performance. In **Noble et al (2016)** the risk was high in the study selection, however low in the evaluation of the endpoints and in the study performance. In **Haukedal et al (2018)** the risk for biases was medium in the selection, low in the selection of the endpoints, however high in the study performance. **Noble et a. (2016)** had the higher quality study design. **Haukedal** **et al. 2018**) had the biggest sample.

**References**

1. Bench J, Kowal A, Bamford J (1997) The BKB (Bamford-Kowal-Bench) sentences lists for partially- hearing children. *Br J Audiol* 13:1–12.
2. Cochrane Deutschland, Arbeitsgemeinschaft der Wissenschaftlichen Medizinischen Fachgesellschaften - Institut für Medizinisches Wissensmanagement. „Bewertung des Biasrisikos (Risiko systematischer Fehler) in klinischen Studien: ein Manual für die Leitlinienerstellung“. 1. Auflage 2016.
3. Haukedal CL, von Koss Torkildsen J, Lyxell B, Wie OB. Parents' Perception of Health-Related Quality of Life in Children With Cochlear Implants: The Impact of Language Skills and Hearing. J Speech Lang Hear Res 2018 8;61:2084-2098. doi: 10.1044/2018_JSLHR-H-17-0278.
4. Hochmair-Desoyer I, Schulz E, Moser L and Schmidt M (1997) The HSM sentence test as a tool for evaluating the speech understanding in noise of cochlear implant users. Am J Otol 18: S83.
5. Huber M (2005) Health-related quality of life of Austrian children and adolescents with cochlear implants. *Int J Pediatr Otorhinolaryngol* 69:1089-1101. [doi.org/10.1016/j.ijporl.2005.02.018](https://doi.org/10.1016/j.ijporl.2005.02.018)
6. Kubba H, Swan IR and Gatehouse S (2004) The Glasgow Children’s Benefit Inventory: a new instrument for assessing health-related benefit after an intervention. *AnnOtol Rhinol Laryngol*. 113(12):980-986. DOI: [10.1177/00034894041130120](https://doi.org/10.1177/000348940411301208)
7. Liberati A, Altman DG, Tetzlaff J, Mulrow C, Gøtzsche PC, Joannidis JPA et al. (2009) The PRISMA statement for reporting systematic reviews and meta-analyses of studies that evaluate healthcare interventions: explanation and elaboration. [PLoS Med 6: e1000100. doi:10.1371/journal.pmed.1000100](http://www.plosmedicine.org/article/info:doi/10.1371/journal.pmed.1000100)
8. Looi V, Lee ZZ and Loo JH (2016b) Quality of life outcomes for children with hearing impairment in Singapore. Int J Pediatr Otorhinolaryngol 80:88-100. DOI: 10.1016/j.ijporl.2015.11.011.
9. Myhrum, M., Tvete, O. E., Heldahl, M. G., Moen, I., & Soli, S. D. (2016). The Norwegian hearing in noise test for children. Ear Hear 37:80-92. doi: 10.1097/AUD.0000000000000224
10. [Noble JH](https://www.ncbi.nlm.nih.gov/pubmed/?term=Noble%20JH%5BAuthor%5D&cauthor=true&cauthor_uid=26756157), [Hedley-Williams AJ](https://www.ncbi.nlm.nih.gov/pubmed/?term=Hedley-Williams%20AJ%5BAuthor%5D&cauthor=true&cauthor_uid=26756157), [Sunderhaus L](https://www.ncbi.nlm.nih.gov/pubmed/?term=Sunderhaus%20L%5BAuthor%5D&cauthor=true&cauthor_uid=26756157), [Dawant BM](https://www.ncbi.nlm.nih.gov/pubmed/?term=Dawant%20BM%5BAuthor%5D&cauthor=true&cauthor_uid=26756157), [Labadie RF](https://www.ncbi.nlm.nih.gov/pubmed/?term=Labadie%20RF%5BAuthor%5D&cauthor=true&cauthor_uid=26756157), [Camarata SM](https://www.ncbi.nlm.nih.gov/pubmed/?term=Camarata%20SM%5BAuthor%5D&cauthor=true&cauthor_uid=26756157) et al. (2016) Initial results with image-guided cochlear implant programming in children. *Otol Neurotol* 37: E63–E69. DOI:[10.1097/MAO.0000000000000909](https://doi.org/10.1097/MAO.0000000000000909)
11. Ravens-Sieberer U, Erhart M, Wille N, Bullinger M, BELLA study group (2008) Health-related quality of life in children and adolescents in Germany: results of the results of the BELLA study. Eur Child Adolesc Psychiatry17 Suppl 1:148-156. doi: 10.1007/s00787-008-1016-x
12. [Spahr AJ](https://www.ncbi.nlm.nih.gov/pubmed/?term=Spahr%20AJ%5BAuthor%5D&cauthor=true&cauthor_uid=24658601), [Dorman MF](https://www.ncbi.nlm.nih.gov/pubmed/?term=Dorman%20MF%5BAuthor%5D&cauthor=true&cauthor_uid=24658601), [Litvak LM](https://www.ncbi.nlm.nih.gov/pubmed/?term=Litvak%20LM%5BAuthor%5D&cauthor=true&cauthor_uid=24658601), [Cook SJ](https://www.ncbi.nlm.nih.gov/pubmed/?term=Cook%20SJ%5BAuthor%5D&cauthor=true&cauthor_uid=24658601), [Loiselle LM](https://www.ncbi.nlm.nih.gov/pubmed/?term=Loiselle%20LM%5BAuthor%5D&cauthor=true&cauthor_uid=24658601), [DeJong MD](https://www.ncbi.nlm.nih.gov/pubmed/?term=DeJong%20MD%5BAuthor%5D&cauthor=true&cauthor_uid=24658601), et al. (2014) Development and Validation of the Pediatric AzBio Sentence Lists. *Ear Hear* 35:418–422. DOI:[10.1097/AUD.0000000000000031](https://doi.org/10.1097/AUD.0000000000000031)
13. Sparreboom M, Snik AF and Mylanus EA (2012) Sequential bilateral cochlear implantation in children: quality of life. *Arch Otolaryngol Head Neck Surg* 138:134-141. DOI: [10.1001/archoto.2011.229](https://doi.org/10.1001/archoto.2011.229)
14. Varni JW, Burwinkle TM and Seid M (2006) The PedsQL 4.0 as a school population health measure: feasibility, reliability, and validity. *Qual Life Res*.15(2):203-215. DOI: [10.1007/s11136-005-1388-z](https://doi.org/10.1007/s11136-005-1388-z)

**Fig. a** PRISMA flowchart showing the process of paper identification and selection.

**
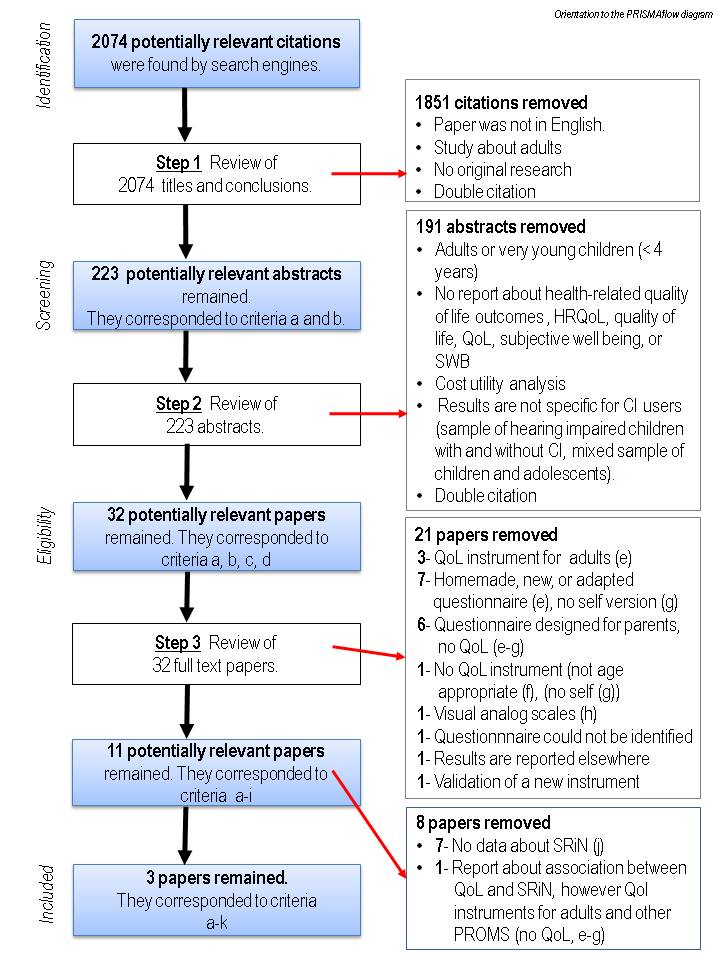
**

**Table a List of inclusion criteria for the review**

1. Original research with n>12 in the study group.
2. The paper (peer reviewed publication) is written in English.
3. Study participants are children and adolescents with CI, with an age range between 4 and 18 years. The mean age of the sample is higher than 5 years.
4. The paper reports about health-related quality of life, HRQoL, quality of life, QoL, subjective well being, or SWB.
5. HRQoL, QoL, or SWB instrument is designed for the use in children and adolescents.
6. HRQoL, QoL, or SWB instrument is age appropriate (versions for very young children, children at school age or for adolescent).
7. HRQoL, QoL, or SWB instrument contains self-reports or self versions.
8. HRQoL instrument: Self-reports and parent-reports are based on physical, mental and social dimensions.
9. The HRQoL, QoL, or SWB instrument is validated^2^ for the age group of children or adolescents addressed in the study.
10. The paper reports about possible associations between speech recognition in noise and QoL of young CI users.
11. Validated audiological speech tests are used for the evaluation of speech perception performance in noise.

*^2^ There is at least one validation study*

| **Author** | **Year** | **Topic** | **Chron. Age, mean** | **Age at 1^st^ CI** | **Correlation QoL chron. Age** | **Corr . QoL,**  **age at 1^st^ CI** |
| --- | --- | --- | --- | --- | --- | --- |
| Percy-Smith et al. | 2008 | SWB | 7y | 4y | / | / |
| Chmiel et al. | 2000 | QoL | 11y | / | / | / |
| Stacey et al. | 2006 | QoL | ? | 2.0-12.6 | / | / |
| Huttunen et al. | 2009 | QoL | 5y | 2y (median) | / | / |
| Schorr et al. | 2009 | QoL | 9y | 3.3 | n.s. | n.s. |
| Hashemi et al. | 2011 | QoL | ? | ? | / | / |
| Edwards et al. | 2012 | QoL | HI 7y, NH 8.1y | 3.8y | / | / |
| Fortunato-Tavares et al. | 2012 | QoL | 6.2y | 4.6y | / | / |
| Sparreboom et al. | 2012 | QoL | Bilateral:  Pre ass: 5y  Post ass 1: 6y  Post ass 2: 7y  Unilateral  Matched by bilateral | Bilateral:  1^th^ CI 1.8y ,  2^nd^ CI 5.3y,  Unilateral:  1.6y | / | n.s. |
|  |  |  |  |  |  |  |
| Almeida et al. | 2015 | QoL | 90.5m | 54.4m | / | / |
| Kumar et al. | 2015 | QoL | 9.9y | 2.5y | n.s. | n.s. |
| Yorgun et al. | 2015 | QoL | 2-18y | ? | / | / |
| Samuel et al. | 2016 | QoL | <5y | 3.7y | / | / |
| Razafimahefa-Raoelina | 2016 | QoL | 10y | 0–1y n= 2  1–2 y n=15  2–3 y n=15 | / | / |
| Noble et al. | 2016 | QoL | 10y | / | / | / |
| Speaker et al. | 2018 | QoL | ? | 46.7m | / | / |
| Sach et al. | 2007 | HRQoL | 9.3 | ? | / | / |
| Lovett | 2010 | HRQoL | Uni 86.4 m, bi 87.0 m, NH 54.2 m | ? | / | / |
| Clark et al. | 2012 | HRQoL | 27m | ? | / | ? |
| Liu et al. | 2016 | HRQoL | 4-11 | ? | / | / |
| Looi et al. (a) | 2016 | HRQoL | HA 10.2y, CI 8y | 62.5 m | / | / |
| Zhao et al. | 2018 | HRQoL | 40.5m | 24.8m | n.s. | n.s. |
| Huber | 2005 | HRQoL | c 10.5y, ad 14.5y | c 4.3y,  ad 7.6y | n.s. | n.s. |
| Loy et al. | 2010 | HRQoL | c 9.1y, ad 13.7y | c 3.3y,  ad 5.8y | / | sig ad:  r=−0.43 (0.01) |
| Warner-Czyz et.al. | 2011 | HRQoL | yc 5.8y,  c 9.1y,  ad 13.7y | yc 2.5y,  c 3.4y,  ad 5.7y | sig:  (r = -0.45, p < 0.0001) | n.s. |
| Meserole et al. | 2014 | HRQoL | CI 87.8m,  NH 8.1m | 2.2y | / | / |
| Duarte et al. | 2014 | HRQoL | CI 11y,  HA 13y,  NH 10y | 2y n= 6,  3y n=10  4y n= 3,  5y n=1 | / | / |
| Looi et al. | 2016 | HRQoL | CI 8y,  HA 10.2y NH 7.2y | 62.5m | / | / |
| Zaidman-Zait et al. | 2017 | HRQoL | With DD 5.3y  Without DD 5.5 y | DD 27.1m  Without DD 23.8m | *Sig.QoL Familiy:*  *r* = −0.42, *p* < .01 QoL Total  *r* = −0.32, *p* < 0.05 | *Sig. Qo School :*  *r* = −0.30, *p* < 0.05). |
| Haukedal et al. | 2018 | HRQoL | 9.2y | Pre deaf  20m, HI 34 m | n.s. | n.s. |

**Table b** Studies addressing quality of life of children and adolescents with CI

***Abbreviation****s****: QoL*** *Quality of Life,* ***HRQoL*** *Health Related Quality of Life,* ***SWB*** *Subjective Well Being,* ***y*** *years,* ***mo*** *months,* ***c*** *children,* ***p*** *parents,* ***t*** *teachers,* ***CI***  *CI users,* ***NH*** *normal hearing,* ***uni-*** *unilateral,* ***bilat*** *bilateral,* ***pre***-*pre (2^nd^) CI,* ***post***-*post (2^nd^) CI,* ***HA*** *hearing aid,* ***DD*** *developmental disability,* ***n.s.*** *not significant,* **sig.** significant, ***pre deaf****-prelingual deaf.*

***Yellow backgroun****d: Paper with validated child centered, and age appropriate QoL, instrument (SWB, HRQoL)*

**Table c** Short checklist for the assessment of risk of bias in studies, included in the review. (In orientation to the Cochrane risk of bias tool, Cochrane Deutschland 2016). Paper a: Huber (2005), paper b: Noble et al 2016, paper c: Haukedal et al. (2018**)**

|  | | **Yes** | | | **No** | | | **Not clear** | | |
| --- | --- | --- | --- | --- | --- | --- | --- | --- | --- | --- |
|  |  | Pr **a** | Pr **b** | Pr **c** | Pr **a** | Pr **b** | Rr **c** | Pr **a** | Pr **b** | Pr **c** |
| 1. **Selection** | |  | | | | | | | | |
| 1. Description of participants: Did the authors provide data about clinical variables, e.g. reasons of hearing loss, years with CI? | yes | yes | yes |  |  |  |  |  |  |  |
| 1. Description of participants: Did the authors provide data about the social background e.g. education of the parents, skill level of the parents, family income? ^†^ |  |  |  |  | no | no | yes^#^ |  |  |  |
| 1. Representativity: Did the author describe the recruiting of the participants? | yes |  | yes |  | no |  |  |  |  |  |
| 1. Representativity: Did the authors inform about the number and reasons of nonparticipation? | yes |  |  |  | no |  |  |  | not clear |  |
| 1. **Endpoints Speech recognition in noise (SRiN), QoL** |  | | | | | | | | |  |
| 1. Did the authors use validated speech tests for the examination of SRiN ? *^‡^* | yes | yes | yes |  |  |  |  |  |  |  |
| 1. If preschool children were participating in the study, are the SRiN tests validated for this age group ? | / | yes | yes | / |  |  | / |  |  |  |
| 1. Did the authors use validated pediatric QoL instruments? *^‡^* | yes | yes | yes |  |  |  |  |  |  |  |
| 1. Did the authors use self reports (additionally to parent reports) | yes | yes |  |  |  | no |  |  |  |  |
| 1. **Study Performance** |  | | | | | | | | |  |
| 1. Does the paper report about time schedules for the SRiN testing, which were identical for all participants of the sample, e.g. in every single case the SRiN tests were performed 6 months after the activation of CI ? |  | yes |  | no |  | no |  |  |  |  |
| 1. Does the paper report about time schedules for the assessment of QoL, which were identical for all participants of the sample, e.g. in every single case the HRQoL assessment was performed 3 years after the activation of CI ? |  | yes |  | no |  | no |  |  |  |  |
| 1. Is the SRiN test procedure described in detail ? |  | yes |  | no |  | no |  |  |  |  |
| 1. Is the QoL assessment procedure described in detail ? |  |  |  | no | no | no |  |  |  |  |
| ***Footnotes****:* ***†*** *Social background may have an impact on the QoL of children with CI (****Looi et al 2016****).* ***‡*** *Item is fulfilled by all papers included in the review (see* ***Table*** *1, criterion e, respective g,h). This Item is listed in the checklist for the sake of completeness.*  ***§*** *See introduction.* ***#*** *Information, that all families belonged to the middle class.*  ***Abbreviation: Pr*** *paper* | | | | | | | | | | |
